# Serum response factor is required for cell contact maintenance but dispensable for proliferation in visceral yolk sac endothelium

**DOI:** 10.1186/1471-213X-11-18

**Published:** 2011-03-14

**Authors:** Mary L Holtz, Ravi P Misra

**Affiliations:** 1 Department of Biochemistry, Medical College of Wisconsin, 8701 Watertown Plank Road, Milwaukee, Wisconsin, 53226, USA

## Abstract

**Background:**

Endothelial-specific knockout of the transcription factor serum response factor (SRF) results in embryonic lethality by mid-gestation. The associated phenotype exhibits vascular failure in embryos as well as visceral yolk sac (VYS) tissues. Previous data suggest that this vascular failure is caused by alterations in cell-cell and cell-matrix contacts. In the current study, we sought to more carefully address the role of SRF in endothelial function and cell contact interactions in VYS tissues.

**Results:**

Tie2-Cre recombinase-mediated knockout of SRF expression resulted in loss of detectable SRF from VYS mesoderm by E12.5. This loss was accompanied by decreased expression of smooth muscle alpha-actin as well as vascular endothelial cadherin and claudin 5, endothelial-specific components of adherens and tight junctions, respectively. Focal adhesion (FA) integrins alpha5 and beta1 were largely unchanged in contrast to loss of the FA-associated molecule vinculin. The integrin binding partner fibronectin-1 was also profoundly decreased in the extracellular matrix, indicating another aspect of impaired adhesive function and integrin signaling. Additionally, cells in SRF-null VYS mesoderm failed to reduce proliferation, suggesting not only that integrin-mediated contact inhibition is impaired but also that SRF protein is not required for proliferation in these cells.

**Conclusions:**

Our data support a model in which SRF is critical in maintaining functional cell-cell and cell-matrix adhesion in endothelial cells. Furthermore, we provide evidence that supports a model in which loss of SRF protein results in a sustained proliferation defect due in part to failed integrin signaling.

## Background

Serum response factor (SRF) is a member of the MADS (MCM1, Agamous, Deficiens, SRF) family of nuclear transcription factors. SRF acts as a dimer to recognize the serum response element (SRE), a ten base pair AT-rich sequence (CC(AT)_6 _GG), also referred to as the CArG box [1,2]. The SRE binding sequence is found in a diverse array of genes including cellular immediate early genes (IEGs), neuronal nuclear receptors, and cytoskeletal and contractile proteins. The specificity of SRF regulatory actions is context dependent and relies on combinatorial interactions between SRF and various accessory factors. The Elk-1 and SAP-1 Ets family members, which form nuclear complexes with SRF, are direct targets for mitogen activated kinase (MAPK) phosphorylation. Also, the myocardin family of SRF-interacting proteins (MRTFs) are important for regulating transcriptional targets associated with Rho-mediated actin polymerization [3].

SRF is a central regulator of myogenic gene expression, cell differentiation and function. It is robustly expressed in cells of myogenic lineage [4-6], and required for differentiation and development of skeletal myoblasts [7,8], cardiomyocytes [9,10] and smooth muscle cells (SMC) [1,11,12]. The expression and regulation of muscle cell contractile proteins depend on SRF transcriptional control [13,14], and SRF has been shown to provide a direct link between alterations in actin dynamics and consequential changes in nuclear transcription (reviewed in [15]. The G-actin associated protein MAL (a.k.a. myocardin-related transcription factor-4, MRTF-4) is released from monomeric actin upon Rho GTP-ase mediated actin polymerization [3]. Once released, MAL translocates to the nucleus and interacts with SRF to mediate gene transcription of cytoskeletal apparatus proteins such as vinculin, actins, myosin, and focal adhesion (FA) molecules as well as SRF itself [16,17].

SRF has also been implicated as an important regulator of numerous events during early development. Embryos globally lacking SRF are unable to generate the embryonic mesoderm germ layer and die during gastrulation [18]. Tissue specific deletions of the *Srf *gene show it is essential for vascular SMC differentiation (reviewed in [12] and cardiogenesis [9,19,20]. SRF is also important for development of brain cells [21,22], immune cells [23] and skin epithelium [24]. A requirement for SRF in early vasculogenesis has been demonstrated by virtue of its importance as a regulator of SMC gene expression. In avian systems, SRF is required for differentiation of coronary SMC from progenitors within the proepicardium, a transient embryonic structure that contributes to coronary vasculogenesis [25,26].

SRF and other members of the MADS-box family have also been shown to regulate cell growth and proliferation in numerous cell types, including rat embryonic fibroblasts [27], myoblasts [28], and gut and liver tissues [29]. While the precise mechanism by which SRF controls proliferation is not known, it has been demonstrated that activated MAPK phosphorylates a nuclear complex containing SRF and Ets/TCFs to induce expression of the IEG *c-fos *[30,31]. Therefore, it is likely that SRF is at least critical for MAPK-mediated cellular proliferation where it acts to mediate cellular IEG expression and enable the G_0 _to G_1 _cell cycle transition [30-33]. SRF is also important for proper cellular adhesion. In particular, several proteins associated with integrin-fibronectin signaling at FA are known SRF-target genes; among them are integrins α1, α5, α9, β1, talin 1, vinculin, and syndecans 2 and 4 [14]. Matrix metalloproteinase 9 (MMP9) is also potentially regulated by SRF [15]. MMP9, together with MMP2, is responsible for degradation of fibronectin and other ECM proteins, suggesting SRF plays a role in the modulation of extracellular matrix (ECM) deposition and maintenance as well.

While SRF has been established as a critical regulator of myogenic cells, relatively little is known about the role of SRF in vascular endothelial cells (VEC). Chai and colleagues [34] showed that SRF is required for appropriate vascular endothelial growth factor (VEGF)-dependent signaling in endothelial cells in vitro, suggesting a role for SRF in VEC function. More recent in vivo studies from our laboratory and others demonstrate that SRF plays a critical role in endothelial cell function during early vascular development in the mouse [35,36]. Knockout of SRF expression in an endothelial specific manner by either Tie2-Cre [36] or Tie1-Cre [35] -mediated genomic recombination resulted in death by embryonic day 13-14.5 (E13-E14.5). TIE2 is a tyrosine kinase receptor expressed specifically on endothelial cells where it acts to mediate angiopoietin signaling [37]. Both studies suggest that the defect in SRF-null VECs stems from dysfunctional cell-cell and cell-ECM contacts. Lack of appropriate cell contacts could also lead to inappropriate vascular permeability (e.g. ions, solutes) as well as gross vascular damage, causing vascular failure and formation of microthrombi [38], which are especially notable in the heart and body of VEC-specific SRF-null embryos [36]. We also observed severe disruption of vascular integrity in visceral yolk sac (VYS) tissues.

In the current study, we sought to characterize the severe disruption of VYS vascular tissues observed in VEC-specific SRF-null embryos. Mice homozygous for a floxed *Srf *gene, *Srf^f/f ^*[39] were bred to mice expressing *Tie2*-promoter driven Cre recombinase [40]. The resulting *Tie2-Cre^+/0 ^Srf^f/f ^*mutant genotype resulted in embryonic lethality by E13.5. Analysis of *Tie2-Cre *construct expression in mid-gestation VYS revealed widespread contributions to VYS mesoderm tissue from early progenitors. Adhesion molecule organization, cell-cell and cell-matrix contacts, and various junction complexes as well as actin dynamics associated with intracellular signaling are disrupted throughout the VYS mesoderm following the Tie2-Cre-mediated loss of SRF. These results are consistent with previous studies suggesting SRF plays a role in controling expression of adhesion molecules and is involved with cell-matrix associated signaling cascades. Additionally, we find that SRF-null VYS mesoderm cells continue to proliferate while wild-type tissues with unimpaired adhesive contacts do not. These data suggest that perturbed signaling through cell-cell and cell-matrix contacts results in a loss of adhesion-dependent growth arrest. These data also suggest that a non-SRF dependent mechanism such as the Jak/STAT pathway may be responsible for proliferation in VYS mesoderm. Consequently, SRF appears to be vital for the formation and maintenance of adhesive contacts but dispensable for proliferation during angiogenic remodeling and vascular plexus maturation.

## Results

### Tie2-Cre-mediated loss of SRF is specific to VYS mesoderm endothelial cells and is complete by E12.5

**Figure 1:**
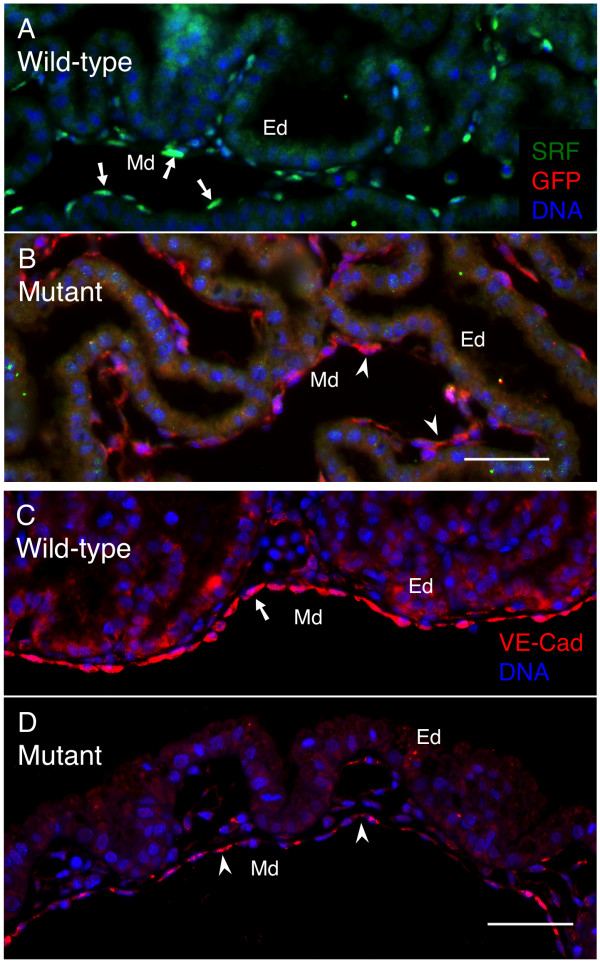
**SRF protein is lost in VYS mesoderm endothelial cells expressing Tie2-Cre recombinase**. Double-label immunofluorescence images of E12.5 VYS tissue from wild type (*Tie2Cre^-/-^·Srf^f/f^*·*ROSA26R-eYFP^+/+^*; A, C) and SRF-null (*Tie2Cre^+/0^·Srf^f/f^*·*ROSA26R-eYFP^+/+^*; B, D) embryos. (A, B) Immunostaining of eYFP by anti-GFP antibody was used to detect evidence of functional Tie2-Cre expression. Tissues in A & B were stained for SRF (green), GFP (red), and DNA (blue). Arrows in A highlight SRF-positive nuclei in VYS mesoderm; arrowheads in B highlight GFP positive cells in VYS mesoderm. (C, D) VE-Cad labels endothelial cells in VYS mesoderm. Tissues in C & D were stained for VE-Cad (red) and DNA (blue). Arrow (C) and arrowheads (D) indicate positive VE-Cad staining. Magnification = 200×, scale bars = 50 μm; Ed = endoderm, Md = mesoderm.

To establish the location and identity of cells in VYS expressing the *Tie2-Cre *construct, indicating which cells would be targeted for *Srf *genomic recombination, we bred male mice expressing the *Tie2-Cre *construct and heterozygous for the floxed *Srf *allele (*Tie2-Cre^+/0^·Srf^f/+^*) to female mice homozygous for both the floxed *Srf *allele and the *ROSA26R-eYFP *fluorescent reporter transgene (*Srf^f/f^·ROSA26R-eYFP^+/+^*). This genetic combination enabled us to track effective Tie2-Cre-mediated excision by staining for eYFP expression, identifying cells in which the floxed *Srf *gene had potentially undergone recombination. Subsequent double-label immunofluorescence analysis of E12.5 VYS revealed that loss of detectable SRF protein was specific to cells with efficient Tie2-Cre activity (see Figure 1A &1B). VYS mesoderm cells expressing Tie2-Cre as shown by the presence of eYFP protein contained no detectable levels of SRF in contrast to comparable *Tie2-Cre^-/- ^*VYS tissues. eYFP expression was observed throughout the VYS mesoderm but not in VYS endoderm. Immunofluorescence detection of vascular endothelial cadherin (CDH5, a.k.a. VE-Cad) demonstrated the endothelial identity of cells within the VYS mesoderm (see Figure 1C &1D). Separate studies using *ROSA26R-β-galactosidase *reporter transgenic mice confirm the observed eYFP expression pattern (see Additional File 1). For these experiments, male *Tie2-Cre^+/0 ^*mice were bred with female *ROSA26R-βgal^+/+ ^*mice; resulting VYS tissues were Lac-Z stained to uncover Tie2-Cre-related β-galactosidase activity. Lac-Z staining was evident in vascular tissues by E10.5; by E12.5 staining was robust and widespread throughout the VYS mesoderm but completely absent from VYS endoderm. Taken together, these data demonstrate the VYS mesoderm endothelial-specific expression of Tie2-Cre.

**Figure 2:**
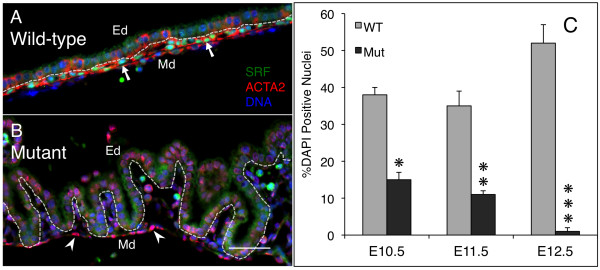
**SRF protein loss is complete by E12.5 in *Tie2Cre^+/0^·Srf^f/f ^*embryos**. Double-label immunofluorescence images of VYS tissue from wild type (A) and SRF-null (B) embryos at E12.5. Tissues were stained for SRF (green), ACTA2 (red), and DNA (blue). Arrows in A highlight SRF-positive nuclei; arrowheads in B mark nuclei lacking SRF. Dashed line indicates division between Ed and Md layers. Magnification = 200×, scale bar = 50 μm; Ed = endoderm, Md = mesoderm. See Additional File 2 for comparable immunofluorescence images from E10.5 and E11.5 tissues. (C) Cytometric analysis of SRF-positive nuclei in E10.5, E11.5, and E12.5 VYS mesoderm cells. Values are expressed as a percentage of total DAPI-stained nuclei counted. SRF protein loss is complete by E12.5. *p = 0.0003; **p = 0.0004; ***p = 0.00008.

The *Tie2-Cre *transgenic construct begins expressing by E7.5 in early VYS in the mouse embryo [40]. This timing coincides with the development of VYS blood islands and onset of initial haematopoiesis [41]. Vascular structures in VYS mature, and blood cell production continues until haematopoiesis shifts to sites within the embryo by E12.5. We previously showed that Tie2-Cre-mediated loss of SRF results in embryo lethality by E13.5 [36]. We showed that *Tie2Cre^+/0^·Srf^f/f ^*embryos appear normal until E10.5 but begin exhibiting evidence of vascular failure and haemorrhaging by E11.5. This becomes more pronounced by E12.5, and embryos are dying or dead by E13.5. VYS tissues from these embryos mirror the same timeframe of vascular disruption as observed in the embryo. The *Tie2-Cre *construct begins expression in VYS at E7.5, some days earlier than a grossly observable phenotype at E11.5. We therefore determined the timeline of SRF loss within the VYS mesoderm by generating embryos using the breeding scheme described above, and harvesting *Tie2Cre^+/0^·Srf^f/f ^*and wild-type littermate embryos at E10.5, E11.5, and E12.5 for SRF expression analysis. We found that SRF loss is complete in VYS mesoderm cells of *Tie2Cre^+/0^·Srf^f/f ^*embryos by E12.5 (see Figure 2A &2B) despite being detectable in both wild type and mutant tissues at E10.5 and E11.5 (see Additional File 2). We counted individual SRF-positive nuclei in VYS mesoderm from *Tie2Cre^+/0^·Srf^f/f ^*and wild-type littermate embryos, and expressed the result as a percentage of total nuclei detected (DAPI stain) (see Figure 2C). The number of VYS mesoderm cells containing detectable levels of SRF is significantly decreased at E10.5 (38 ± 2% WT vs. 15 ± 2% *Tie2Cre^+/0^·Srf^f/f^*; p = 0.0003), as well as at E11.5 (35 ± 4% WT vs. 11 ± 1% *Tie2Cre^+/0^·Srf^f/f^*; p = 0.0004) and nearly absent from E12.5 *Tie2Cre^+/0^·Srf^f/f ^*VYS mesoderm (52 ± 13% WT vs. 1 ± 1% *Tie2Cre^+/0^·Srf^f/f^*; p = 0.00008). To establish that the lowered levels of SRF protein were capable of affecting the expression of SRF-dependent genes, tissues stained for SRF were also stained for smooth muscle α-actin (ACTA2). ACTA2 is a cytoskeletal protein dependent on SRF for expression [42] and was not used as a marker for the presence of smooth muscle cells. ACTA2 expression was detectable in wild-type VYS mesoderm by E10.5 and remained so through E12.5 (see Figure 2 and Additional File 2), but appeared disrupted in E10.5 *Tie2Cre^+/0^·Srf^f/f ^*VYS tissues. Disruption of ACTA2 expression continued and worsened in E11.5 and E12.5 *Tie2Cre^+/0^·Srf^f/f ^*VYS mesoderm.

Together the data presented in Figures 1 and 2 demonstrate that SRF protein is progressively lost from E10.5 through E12.5, being largely or completely lost by E12.5 in VYS mesoderm tissues of *Tie2Cre^+/0^·Srf^f/f ^*embryos. Complete loss of SRF protein is preceded by detectable alterations in SRF-dependent proteins such as ACTA2.

### Alterations in cell contacts contribute to VYS failure

SRF has been demonstrated to be an important regulator of cell shape, integrity, migration, and adhesion (reviewed in [15]. Cytoskeletal elements rely on interactions with the plasma membrane to transduce extracellular signals generated by receptor ligands and ECM proteins. We previously showed ultrastructural evidence that loss of SRF in VYS tissues resulted in a lack of cell-cell adhesion contacts and disrupted ECM deposition [36]. Using electron microscopy analysis of E12.5 VYS from *Tie2Cre^+/0^·Srf^f/f ^*embryos, we found cells in VYS mesoderm lacked appropriate cell-cell junctions compared to wild-type littermate tissues. We also observed deficient ECM deposition between mesoderm and endoderm layers in these same tissues. Our observations are consistent with other studies that point to a role for SRF in regulation of adhesion molecules. Embryonic stem cells lacking SRF are unable to form FAs or bind appropriately with different ECM components [43]. Furthermore, expression of FA molecules such as vinculin and tropomyosin is regulated by SRF and MRTFs through a Rho/MAL-dependent mechanism [17]. In the current study, we investigated the cause of the observed loss of tissue integrity in VYS tissues, focusing on the role of SRF in: 1) cell-cell contacts, 2) cell-matrix contacts, and 3) ECM deposition.

To address potential changes in expression of adhesion molecules and ECM in SRF-null VYS mesoderm in more detail, we generated endothelial-specific SRF-null embryos using the breeding scheme described above and harvested *Tie2Cre^+/0^·Srf^f/f ^*and wild-type littermate embryos at E12.5 for analysis. We focused our attention on tissues of this age since the vascular phenotype observed was most consistent by this developmental timepoint. We examined the EC-specific adhesion molecule VE-Cad as a measure of appropriate cell-cell contact. VE-Cad is a VEC-specific transmembrane adhesion protein associated with adherens junctions (AJ) [44]. It forms homodimeric complexes between adjacent cells and is expressed in all VECs upon committed differentiation. *Tie2Cre^+/0^·Srf^f/f ^*VYS mesoderm tissues display decreased VE-Cad immunoreactivity compared to wild-type tissues (see Figure 1C &1D). This result is consistent with our previous observation of contact deficiencies at an ultrastructural level between endothelial cells.

**Figure 3:**
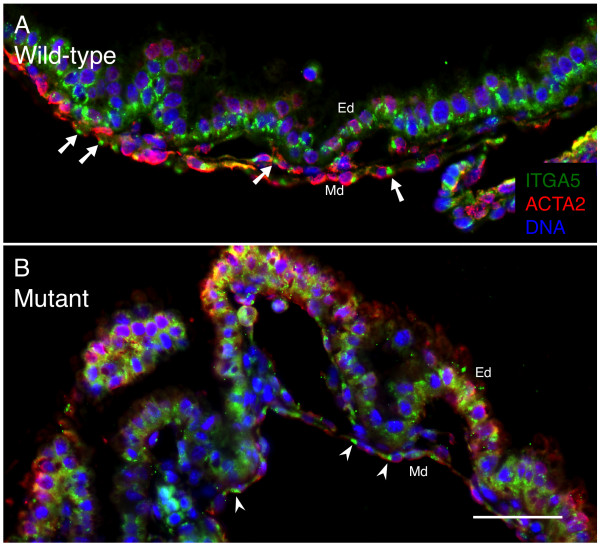
**ITGA5 is maintained despite loss of SRF expression**. Cell-ECM contacts occur at FA consisting of integrin proteins. Double-label immunofluorescence images of VYS tissue from wild type (A) and SRF-null (B) embryos at E12.5. Tissues were stained for ITGA5 (green), ACTA2 (red), and DNA (blue). ACTA2 was used to monitor SRF status in mesoderm due to antibody incompatibility between anti-SRF and anti-ITGA5 antibodies. Arrows and arrowheads highlight points of ITGA5 expression in VYS mesoderm cells. Magnification = 200×, scale bar = 50 μm; Ed = endoderm, Md = mesoderm.

To address cell-matrix contact, we examined integrin α5 (ITGA5, a.k.a. α5) expression. α5 is a member of the integrin family of proteins that form heterodimeric complexes composed of one alpha and one beta chain [45]. α5 pairs with integrin β1 (ITGB1, a.k.a. β1) to bind specifically to fibronectin-1 (FN1) in ECM and form cell-matrix FAs. VECs express several integrin family members, and global knockouts of either α5 or β1 cause defective vascular development and embryonic lethality [46,47]. However, recent studies examining VEC-specific loss of α5 did not detect impaired vasculogenesis [48] in contrast to VEC-specific ablation of β1 that results in embryonic lethality [49]. Other studies suggest that the *Itga5 *gene may be under SRF regulatory control as evidenced by the presence of an SRE within its promoter [50]. We detected α5 protein at sites of cell-matrix interactions in both wild type and *Tie2Cre^+/0^·Srf^f/f ^*VYS tissues (see Figure 3). We did not observe large changes in α5 protein expression patterns despite the lack of SRF protein observed in VYS tissues at this time, suggesting that loss of cell-ECM contacts in SRF deficient animals is due to another mechanism.

**Figure 4:**
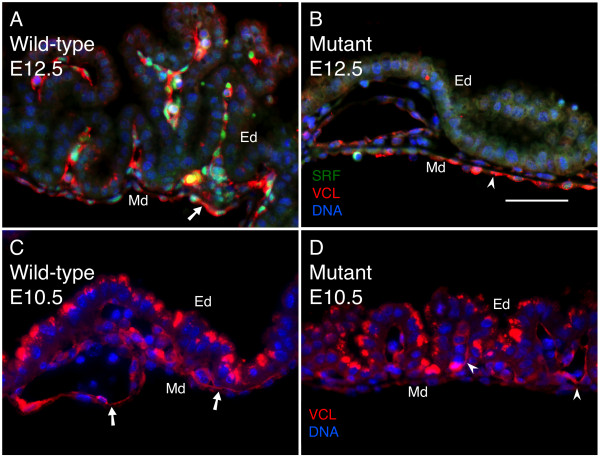
**VCL is moderately disrupted following loss of SRF**. FA-associated intracellular signaling requires VCL interactions between integrins and the actin cytoskeleton. Double-label immunofluorescence images of VYS tissue from wild type (A, C) and SRF-null (B, D) embryos at E12.5 (A, B) and E10.5 (C, D). Tissues were stained for SRF (green), VCL (red), and DNA (blue). Arrows/arrowheads mark VCL protein. Magnification = 200×, scale bar = 50 μm; Ed = endoderm, Md = mesoderm.

To address whether loss of SRF may affect signaling associated with FAs, we examined vinculin (VCL) expression. VCL is a membrane-associated protein involved with linkage of β1 cytoplasmic tails to the actin cytoskeleton [51]. It is important as a regulator of mechanical stress between extracellular forces and intracellular response through cytoskeletal dynamics. VCL expression is dependent on SRF/MRTF-mediated transcriptional regulation, and the *Vcl *gene is predicted to contain an SRE within its promoter [50]. We examined VCL protein expression patterns in VYS mesoderm by immunofluorescence and found detectable protein in both wild-type and SRF-null tissues at E10.5 (See Figure 4C &4D). However, by E12.5, VCL staining was moderately disrupted in *Tie2Cre^+/0^·Srf^f/f ^*compared to wild-type tissues (see Figure 4A &4B). Disturbance of VCL staining was correlated with loss of SRF protein as shown by double-label analysis.

**Figure 5:**
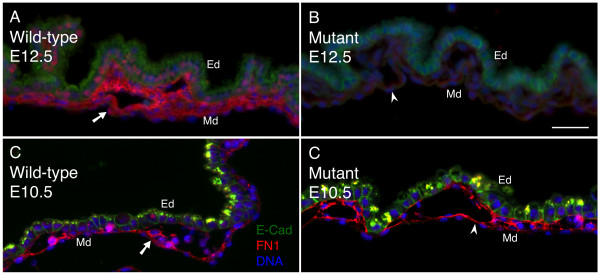
**ECM in *Tie2Cre^+/0^·Srf^f/f ^*VYS tissue lacks FN1**. FN1 contributes to ECM surrounding developing vessels and is critical for signaling in VYS tissues. Double-label immunofluorescence images of VYS tissue from wild type (A, C) and SRF-null (B, D) embryos at E12.5 (A, B) and E10.5 (C, D). Tissues were stained for E-Cad (green), FN1 (red), and DNA (blue). Arrows/arrowheads mark FN1 expression. Magnification = 200×, scale bar = 50 μm; Ed = endoderm, Md = mesoderm.

To address whether SRF plays a role in the loss of ECM deposition we previously observed in *Tie2Cre^+/0^·Srf^f/f ^*VYS tissues, we assayed for the ECM component FN1. FN1 displays robust expression around developing vessels [52] and induces intracellular signaling via interactions with the FN1 receptor integrin pair α5β1 [45]. FN1 protein expression was visible in both wild-type and SRF-null VYS tissues at E10.5 (see Figure 5C &5D). In contrast, *Tie2Cre^+/0^·Srf^f/f ^*VYS tissues showed a profound decrease in detectable FN1 compared to wild-type tissues at E12.5 (see Figure 5A &5B). Tissue sections were co-stained with E-cadherin (CDH1, a.k.a. E-Cad) to demonstrate contact deficient status of SRF-null tissues due to primary antibody incompatibility with the anti-SRF antibody.

Taken together, these data provide evidence that the loss of SRF in VYS mesoderm results in disruption of cell-cell and cell-ECM contacts. Decreased VE-Cad protein indicates that intercellular adhesion between EC is disordered. Cell-ECM adhesion is also disturbed due to a significant lack of ECM-associated FN1 despite apparently unaffected levels of α5. Furthermore, the observed decrease in VCL protein suggests that VCL-dependent intracellular signaling cascades may be perturbed.

### Gene expression analysis shows SRF-null VYS tissue is contact deficient

**Figure 6:**
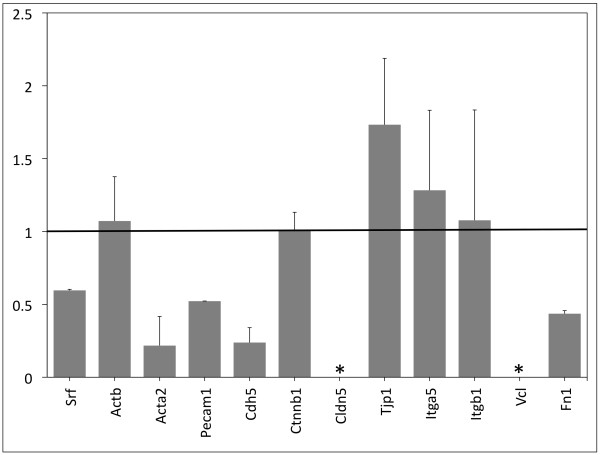
**Adhesion molecule gene expression is disrupted in SRF-null VYS tissues**. Quantification of mRNA levels in whole VYS tissues from wild type and *Tie2Cre^+/0^·Srf^f/f ^*embryos. Fold change was calculated using 2^ΔΔC_t_, where a value of '1' indicates no change. Bars represented as * indicate no signal was detected in samples from *Tie2Cre^+/0^·Srf^f/f ^*embryos. Values represent fold change with positive SEM only. Genes listed: serum response factor (*Srf*), β-actin (*Actb*), smooth muscle α-actin (*Acta2*), platelet-endothelial adhesion molecule-1 (*Pecam1*), vascular endothelial cadherin (*Cdh5, a.k.a. VE-Cad*), β-catenin (*Ctnnb1*), claudin 5 (*Cldn5*), tight junction protein 1 (*Tjp1, a.k.a. ZO-1*), integrin-α5 (*Itga5*), integrin-β1 (*Itgb1*), vinculin (*Vcl*), fibronectin-1 (*Fn1*).

To examine potential changes in gene transcription in SRF-null VYS tissue, we assayed whole VYS from E12.5 embryos using real-time quantitative PCR (qPCR) (See Figure 6). Separation of mesoderm from endoderm in *Tie2Cre^+/0^·Srf^f/f ^*VYS was not feasible due to the fragile nature of SRF-null VYS tissues. We focused our analysis on genes involved in cellular adhesion that are either known or predicted to be targets of SRF regulation as well as those we found altered during our immunofluorescence studies. Results are expressed using the 2^ΔΔC_t _method where a value of 1 indicates no difference between groups. *Srf *expression was found to be decreased (0.596) in *Tie2Cre^+/0^·Srf^f/f ^*VYS tissue; message in VYS endoderm not affected by Tie2-Cre-mediated genomic excision may account for the message detected given the lack of visible SRF signal in VYS mesoderm identified by immunofluorescence. We found a trend toward decreased expression of *Acta2 *(0.215). Our own observations of virtually undetectable expression of ACTA2 protein in SRF-null VYS (Figure 2) support previously reported observations that *Acta2 *expression is SRF-dependent. AJ-associated *VE-Cad *(a.k.a. *Cdh5*) expression was profoundly decreased (0.238), as was expression of another EC-specific homophilic adhesion molecule, *Pecam1 *(0.522). Expression of the intracellular signaling molecule β-catenin (*Ctnnb1*) that partners with VE-Cad and PECAM1 proteins was not changed (1.007). Expression of the EC-specific tight junction (TJ) component claudin 5 (*Cldn5*) was not detectable in mutant tissues, while expression of its associated intracellular signaling molecule tight junction protein 1 (*Tjp1*, a.k.a. ZO1) was slightly increased (1.733). We assayed *Itga5 *and *Itgb1 *expression as a measure of FA assembly and found they were relatively unchanged (1.283 and 1.077, respectively), consistent with our observation of minimal change in α5 protein. In contrast, *Vcl *expression was not detectable in mutant tissues, suggesting that failure of FAs is due at least in part to the lack of linkage between membrane-associated integrins and the actin cytoskeleton. We did not detect any evidence of integrin-linked kinase (*Ilk*) expression in any tissue; *Ilk *qPCR primer specificity was verified against HeLa cell cDNA (data not shown). Furthermore, expression of *Fn1 *was decreased (0.436), implying a lack of ECM substrate for binding of integrins at the plasma membrane. Our immunofluorescence analysis demonstrating profound disruption of both VCL and FN1 proteins corroborates these changes in gene expression. Taken together, expression analysis of the three pairs of junction related proteins suggests a cumulative effect of adhesion failures, and that cell-cell and cell-ECM contact deficiency and impaired integrin signaling is at least partly responsible for the vascular phenotype observed in *Tie2Cre^+/0^·Srf^f/f ^*embryos.

### SRF is not required for proliferation of VYS mesoderm cells

**Figure 7:**
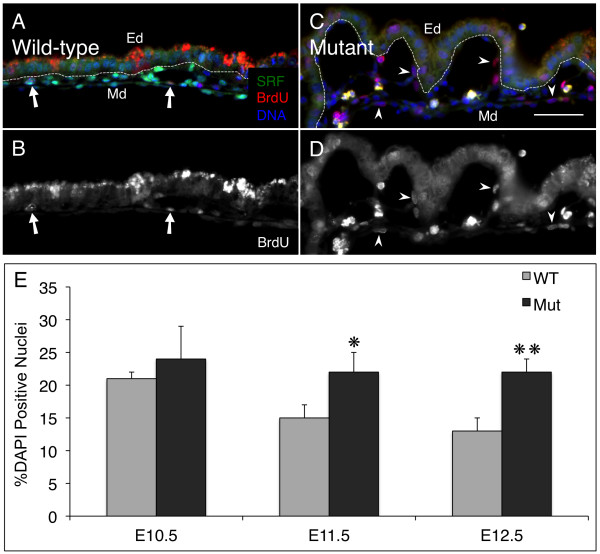
**SRF-null VYS mesoderm tissues show aberrant proliferation**. Immunodetection of BrdU incorporation was used to determine the proliferation status of VYS tissues. Double-label immunofluorescence images of VYS tissue from wild type (A, B) and SRF-null (C, D) embryos at E12.5. Tissues were stained for SRF (green), BrdU (red), and DNA (blue); monochrome images in B and D show BrdU staining in isolation. Arrows in A and B highlight SRF-positive nuclei that colocalize with BrdU; arrowheads in C and D mark nuclei lacking SRF that stain positively for BrdU. Dashed line indicates division between Ed and Md layers. Magnification = 200×, scale bar = 50 μm; Ed = endoderm, Md = mesoderm. (C) Cytometric analysis of BrdU-positive nuclei in E10.5, E11.5 and E12.5 VYS mesoderm. Values are expressed as a percentage of total DAPI-stained nuclei counted. *p = 0.025; **p = 0.007.

**Figure 8:**
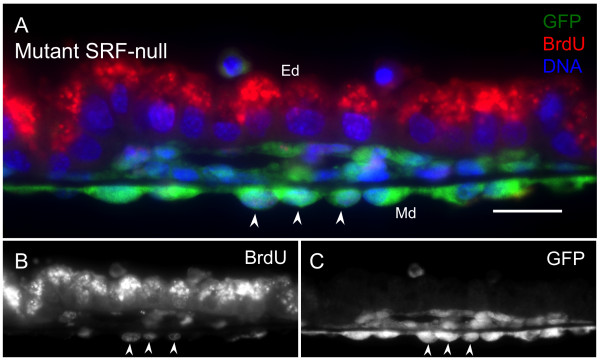
**Tie2Cre-mediated eYFP expression indicates BrdU-positive cells are endothelial**. E12.5 VYS mesoderm tissues from *Tie2Cre^+/0^·Srf^f/f ^*embryos are virtually devoid of SRF protein yet continue to proliferate. Double-label immunofluorescence images of E12.5 VYS from *Tie2Cre^+/0^·Srf^f/f^*·*ROSA26R-eYFP^+/+ ^*embryos. Tissues were stained for GFP (green), BrdU (red), and DNA (blue); monochrome images show anti-BrdU (B) and anti-GFP (C) staining in isolation. Arrows mark individual BrdU-positive cells in VYS mesoderm also expressing eYFP. Magnification = 400×, scale bar = 20 μm; Ed = endoderm, Md = mesoderm.

Consistent with a role for SRF in mediating growth factor signaling, it has been shown in a number of cell types that loss of SRF inhibits proliferation [53]. Cell adhesion-associated signaling is intimately connected to appropriate regulation of proliferation as well as other functions such as migration and differentiation (for review see [54]. These observations together with our experimental results that loss of SRF leads to impaired adhesion initially suggested to us that in SRF-deficient animals, loss of adhesive signals would result in an inhibition of cell proliferation. To address directly whether the adhesive failure of VYS mesoderm vascular tissues observed in SRF-null *Tie2Cre^+/0^·Srf^f/f ^*yolk sac affected proliferation, we assayed the proliferative status of VYS mesoderm cells by BrdU labeling. Timed-pregnant mice at E10.5, E11.5 and E12.5 were treated with BrdU 2-3 hours prior to harvesting embryonic VYS tissues. An anti-BrdU antibody was used to detect the presence of BrdU in nuclei of cells in S phase. BrdU-positive nuclei were counted and results expressed as a percentage of total nuclei detected as before. Surprisingly, we found proliferating cells in both wild-type and SRF-null VYS mesoderm for all time-points examined (see Figure 7). All BrdU-positive cells detected in wild-type tissues also expressed SRF, although most SRF-positive cells were not positive for BrdU demonstrating that SRF expression alone is not sufficient for proliferation. As shown in Figure 7 wild-type VYS mesoderm cells exhibit a decrease in proliferation from E10.5 to E12.5; however, SRF-null VYS mesoderm did not follow this same pattern, showing instead a sustained rate of proliferation. This difference was first observed at E10.5 (21 ± 1% WT vs. 24 ± 5% *Tie2Cre^+/0^·Srf^f/f^*; p = NS), reached statistical significance by E11.5 (15 ± 2% WT vs. 22 ± 3% *Tie2Cre^+/0^·Srf^f/f^*; p = 0.03), and remained divergent at E12.5 (13 ± 2% WT vs. 22 ± 2% *Tie2Cre^+/0^·Srf^f/f^*; p = 0.005). Cells with BrdU-positive nuclei were visible throughout E12.5 VYS mesoderm despite the complete lack of SRF by this time-point. Furthermore, the few remaining SRF-positive cells at E12.5 were all BrdU-positive, even though the vast majority of BrdU-positive cells lacked SRF. It is important to note that the non-nuclear staining observed within the brush-border of the VYS endoderm is a result of anti-mouse secondary antibody detection of maternofetally transferred IgG molecules [55,56]. This phenomenon is confined to the VYS endoderm that functions, in part, to take up IgG molecules secreted into the uterine lumen. No such non-nuclear staining is detected within the VYS mesoderm that is the focus of our analysis. The aberrant proliferation observed in mutant VYS using BrdU analysis was substantiated by similar findings using phosphorylated histone H3 (PhH3) detection to assess proliferation (see Additional File 3). PhH3 is a marker for the heavily phosphorylated form of histone H3 associated with cells in metaphase, and is lost as cells exit the cell cycle. In contrast, BrdU incorporation may be found in DNA synthesized as cells enter the cell cycle, indicating both current and accumulated proliferation since the time of BrdU administration. To verify that these cells were SRF-deficient owing to Tie2-Cre-mediated recombination, we also stained these tissues for eYFP to detect effective Cre recombinase activity (see Figure 8). We found that BrdU-positive cells in *Tie2Cre^+/0^·Srf^f/f ^*VYS mesoderm also expressed eYFP, indicating that their SRF-null status was due to Tie2-Cre-mediated genomic recombination. TIE2 along with VE-Cad have been used extensively to label VECs owing to their restricted expression patterns. VE-Cad is an endothelial-specific adhesion molecule associated with adherens junctions (for review see [57], while TIE2 is a tyrosine kinase receptor for the angiopoietin family of vascular signaling molecules and is expressed by endothelial cells as well as some hematopoietic progenitor cells in early development [58]. Our staining of VYS mesoderm with the endothelial-specific marker VE-Cad confirms the endothelial nature of these proliferative SRF negative cells (See Figure 3A). Taken together with Tie2Cre-excised eYFP expression, the detection of these endothelial markers throughout the VYS mesoderm demonstrate that all proliferative cells detected are endothelial in nature.

To assess the possibility that SRF-null cells were not being detected due to apoptotic loss, we also assayed SRF-null and wild-type littermate VYS tissues using terminal deoxynucleotide transferase dUTP nick end labeling (TUNEL). *Tie2Cre^+/0^·Srf^f/f ^*VYS tissues stained using TUNEL did not show any consistent differences in the level of apoptosis between wild-type and SRF-null tissues, (see Additional File 4), strongly suggesting that the vascular failure observed in SRF null embryos is not due simply to loss of cells in developing VYS.

## Discussion

**Figure 9:**
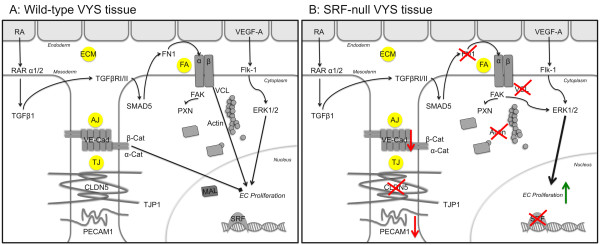
**Loss of SRF results in contact deficiency**. Graphic highlighting the adhesive contacts involved in VYS function and signaling between endoderm and mesoderm tissues. Molecules affected by the loss of SRF have been marked by a red "X" (B). Extracellular matrix (ECM) containing fibronectin-1 (FN1) provides structural support and signal induction via interactions with integrins (α β) at focal adhesions (FA). FN1 is deposited into the ECM in by the mesoderm in response to autocrine transforming growth factor β (TGFβ) signals induced by retinoic acid (RA) released by the endoderm. FAs link intracellularly with the actin cytoskeleton via vinculin (VCL) and to signal cascades such as focal adhesion kinase (FAK). Adherens junctions (AJ) containing vascular endothelial cadherins (VE-Cad, CDH5) and tight junctions (TJ) consisting of claudin 5 (CLDN5) molecules provide cell-cell contacts. PECAM1 molecules provide additional intercellular contacts. Nuclear localized SRF responds to actin dynamics via interactions with MAL when released from monomeric actin. Vascular endothelial growth factor (VEGF) signals via Flk-1 to induce ERK1/2-mediated SRF-dependent proliferation. TJP1 = tight junction protein 1, a.k.a. ZO1; α-Cat = α-catenin, CTNNA1; β-Cat = β-catenin, CTNNB1; PXN = paxillin; RAR1/2 = RA receptors 1, 2; TGFβRI/II = TGFβ receptors I, II.

VYS is comprised of two layers: a mesoderm-derived layer of narrow elongated cells and an endoderm-derived layer of brush border-presenting columnar epithelial cells. VYS mesoderm generates blood cell and vascular precursors within blood islands by E7.5 [59], followed by expansion and maturation of the VYS vascular tree by E13.5. Angiogenic remodeling of initial blood vessels into a mature and efficient vascular plexus requires the orchestration of EC migration, proliferation and apoptosis. Once blood flow begins, hemodynamic stresses cause alterations in adhesion molecule expression profiles and EC function, ultimately leading to vessel rearrangement. These processes depend heavily upon and in turn profoundly affect cellular adhesion, both with other cells and the surrounding matrix (see Figure 9). EC-ECM contact occurs at FAs that consist of integrin receptor pairs and associated intracellular factors. FN1 is a prominent component of the ECM surrounding developing vessels, and it serves as a ligand for the α5β1-integrin receptor pair on EC [60]. FN1-mediated activation of α5β1 leads to the induction of focal adhesion kinase (FAK) signaling pathways including Src recruitment, PI3 kinase induction, Raf/MEK/ERK induction and NF-κB activation (reviewed in [61]. FA proteins such as VCL link α5β1 receptor pairs intracellularly with the actin cytoskeleton [51], allowing for transduction of extracellular signals that impose on actin dynamics. Cell-cell contact between EC occurs at AJ and TJ composed of VE-Cad homodimers and CLDN5 transmembrane proteins, respectively [62]. Both VE-Cad and CLDN5 are EC-specific components of cell-cell adhesive contacts. Each junction type includes intracellular partners that transduce signals caused by extracellular events. These include catenin proteins α- and β-catenin (CTNNA, CTNNB1) in the case of VE-Cad, and ZO-1 associated with CLDN5. Furthermore, activation of integrin-mediated Src signaling results in disruption of VE-Cad-associated AJ, adding to the complex interconnected nature of cellular adhesion [63]. Collectively, the signaling pathways initiated at junctional complexes act to balance the control of cellular proliferation as well as migration (Figure 9). Loss of proteins along these signaling cascades may result in unintended cellular quiescence or uncontrolled proliferation.

Examination of the Tie2-Cre-mediated knockdown of SRF in VYS mesoderm has provided evidence to support a significant role for SRF in endothelial cell function. Previous work demonstrated that endothelial-specific ablation of SRF protein in mice resulted in loss of vascular integrity and function within VYS and ultimately caused embryonic death by mid-gestation [36]. Our current studies suggest that failure of SRF-null VYS tissues and subsequent vascular failure of embryos is due to disrupted junction complexes and junction-related signaling molecules in endothelial cells of the VYS mesoderm. Our observation that loss of SRF leads to alterations in cell contact proteins is consistent with previous reports in other cell types. Embryonic stem cells lacking SRF are unable to form FA or bind appropriately with extracellular matrices [43,64]. Studies in epithelial cells have verified a need for SRF in establishing cell-cell contacts in developing epidermis [24]. Various studies suggest that this effect is dependent at least in part on SRF's ability to regulate actin dynamics, including those necessary for formation and maintenance of adhesive contacts. Actin filaments link with FA via integrin-associated proteins such as α-actinin, talin, and VCL, providing adhesion-related cellular input and subsequent modulation of cell shape, motility, survival and proliferation [65]. Our results demonstrating a lack of VCL in SRF-null tissues strongly suggest that FAs are unable to connect with the actin cytoskeleton appropriately, resulting in impaired FA-dependent signaling.

We detected profound alterations in VCL and ACTA2 protein patterns and mRNA expression levels in SRF-null VYS tissues. Expression of VCL and other FA molecules such as tropomyosin is regulated by SRF and MRTFs through a Rho/MAL-dependent mechanism [17]. Furthermore, the *Vcl *gene has a predicted SRE binding element [50], raising the likelihood that SRF directly controls *Vcl *expression in VYS tissues. Disrupted *Vcl *expression causes perturbations in integrin-actin signaling as well as decreased FAK signaling [43]. *Acta2 *is regulated by SRF, and its loss likely contributes to the defect generated by disrupted VCL, compounding the mis-regulation of SRF-dependent actin dynamics. Our study did not detect significant disruption of *Itga5 *expression despite the presence of a predicted SRE in the *Itga5 *gene [50], perhaps indicating that *Itga5 *is not under SRF regulatory control in VYS tissues. Additionally, there is no evidence to suggest *Fn1 *is under direct regulation by SRF. However, we observed substantial loss of FN1 in ECM of SRF-null VYS tissues, suggesting defects in either the transmission of extracellular signaling or in ECM maintenance. VYS endoderm signals to VYS mesoderm through a retinoic acid-transforming growth factor β1 (TGFβ1) dependent pathway [66], and chimaeric mice lacking functional TGFβ receptors show deficient deposition of FN1 between the VYS endoderm and mesoderm layers [67]. Our observed loss of FN1 protein was accompanied by a slight decrease in *Tgfb1 *expression (data not shown), highlighting the requirement of this signaling cascade for proper VYS development and subsequent vascular integrity. Furthermore, FN1 deposition and maintenance depends on appropriate FA signaling. The lack of intracellular VCL we observed in SRF-null VYS tissues suggests that impaired FA signaling contributes to the loss of extracellular FN1 [68]. Our observation of decreased VE-Cad and CLDN5 proteins suggests that AJ and TJ functions are also impaired. VE-Cad associated with AJ has been demonstrated to mediate contact inhibition of EC proliferation [69]. CLDN5 has not been shown to act on cell cycle progression; however, expression of CLDN5 is at least in part regulated by VE-Cad [70], and suggests that TJ act along with AJ to stabilize EC intracellular adhesion.

**Figure 10:**
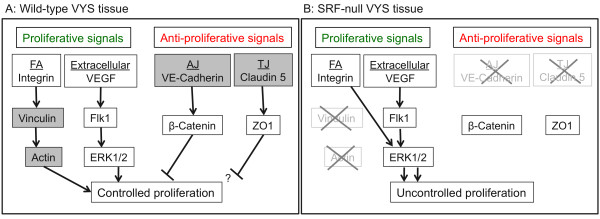
**Model for how loss of SRF-dependent adhesion molecules leads to increased proliferation**. (A) Adhesion-mediated signaling pathways associated with control of proliferation. SRF regulated molecules are shown in grey boxes. (B) Molecules downregulated by the loss of SRF have been faded and marked by an "X". AJ = adherens junction; FA = focal adhesion; TJ = tight junction (see text for further discussion).

Our analysis also revealed that SRF-null VYS endothelial cells have impaired proliferative control, and, in contrast to cells in wild-type VYS, fail to downregulate proliferation. This is consistent with a model in which, during normal development, adhesive contacts and associated signal pathways balance proliferative signals, but that loss of SRF leads to disruption of inhibitory pathways and uncontrolled proliferation (see Figure 10). Our observed loss of VE-Cad is consistent with a loss of the proliferative control conferred by AJ-mediated signaling. It should be pointed out that our model does not posit that SRF serves as a direct inhibitor of proliferation genes, but rather that in some cell types, such as VYS mesoderm endothelial cells, inhibitory pathways are modulated by SRF. Furthermore, unlike in some other cell types, our data provides strong evidence that these cells do not require SRF protein for proliferation, implying utilization of non-SRF-dependent pathways for proliferation.

Given results presented here that SRF-null VYS endothelial cells continue to proliferate, it seems likely that these cells do not require SRF to proliferate even in wild-type tissues. Numerous cell types have demonstrated a dependence on SRF-driven IEG expression for proliferation, including fibroblasts [27] and cells of myogenic lineage [28]. However, few cell types are known to proliferate without SRF, among them mouse embryonic stem cells [64]. Recently, Koegel and colleagues [71] found that hyperproliferative skin cells down-regulate SRF expression, suggesting that there may be multiple cell types that use alternative non-SRF dependent pathways to regulate proliferation. They also observed defects in both cell-cell and cell-matrix adhesive contacts, suggesting that these phenomena are likely interrelated. Furthermore, recent studies of SRF-null hematopoietic stem cells (HSC) demonstrate that they also exhibit cell-cell and cell-matrix adhesion failure, that this failure is due to impaired integrin-related signaling, and that HSC proliferation is not negatively affected by the loss of SRF [72]. These observations are consistent with our model where SRF acts as a modulator of cell-cell and cell-matrix adhesion molecules that indirectly affect proliferation due to a loss of adhesion-mediated inhibition. Down-regulation of SRF in this context allows modulation of adhesive integrity and associated actin-dependent junction maintenance necessary for cellular proliferation and migration; however, SRF-independent proliferation of cells within the VYS mesoderm that is required for angiogenic remodeling remains functionally intact.

## Conclusions

Our study provides evidence to suggest that the role of SRF in proliferation is balanced with its role in actin-dependent junction dynamics. It remains to be determined which junction proteins are affected through direct SRF-mediated transcriptional regulation and which may be dependent on SRF-related actin dynamics for proper function. The intimacy between the actin cytoskeleton and membrane bound adhesion molecules provides a broad reach for SRF to respond to signaling that initiates at the plasma membrane. Our results demonstrating that loss of cellular adhesive contacts results in impaired proliferative control is consistent with previous studies; however, we show for the first time evidence that VYS mesoderm endothelial cells do not require SRF for proliferation. Further study will be necessary to determine which proliferative pathway dominates in these cells.

## Methods

### Mice and genotyping

*Tie2-Cre^+/0 ^*and *Srf^f/f ^*mice have been described previously [39,40]. The *ROSA26R-eYFP^+/+ ^*(stock #006148) and *ROSA26R-βGal^+/+ ^*(stock #003310) reporter mouse strains were purchased from the Jackson Laboratory. Embryos were generated by timed mating, designating noon on the day a vaginal plug was observed as embryonic day 0.5 (E0.5). Genotyping was performed using standard protocols utilizing genomic DNA isolated from embryonic yolk sac, amnion or tail tissue. Primers used were: *Tie2-Cre^+/0 ^*fwd 5'-GTTCGCAAGAACCTGATGGACA-3' and rvs 5'-CTAGAGCCTGTTTTGCACGTTTC-3'; *Srf^f/f ^*fwd 5'-TGCTTACTGGAAAGCTCATGG-3' and rvs 5-'TGCTGGTTTGGCATCAACT. *ROSA26R-eYFP *mice were genotyped according to protocols from Jackson using the following three primers: WT-fwd 5'-GGAGCGGGAGAAATGGATATG-3', eYFP fwd 5'-AAGACCGCGAAGAGTTTGTC-3', and rvs 5'-AAAGTCGCTCTGAGTTGTTAT-3'. *ROSA26R-βGal^+/+ ^*mice were genotyped according to protocols from Jackson using the following three primers: F150 fwd 5'-GGCTTAAAGGCTAACCTGATGTG-3', Tg rvs 5'-GCGAAGAGTTTGTCCTCAACC-3', and WT-rvs 5'-GGAGCGGGAGAAATGGATATG-3'. Animals designated for BrdU incorporation studies were administered BrdU Labeling Reagent by intraperitoneal injection (dose 1 mL/100 g body weight; Invitrogen 00-0103) and harvested for tissues after 2-4 hours. All procedures were in compliance with the Institutional Animal Care and Use Committee of the Medical College of Wisconsin.

### Histology and imaging

Embryos were harvested from timed pregnant females, and yolk sac tissues were dissected and fixed in either Tris-buffered zinc fixative (0.1 M Tris pH7.4 with 3.2 mM calcium acetate, 22.8 mM zinc acetate, and 36.7 mM zinc chloride) or zinc formalin (Richard-Allan Scientific). Samples were processed for paraffin embedding, and sections (5-7 μm) were used for immunofluorescence histochemistry as described [36,73]. Reagents used were: anti-serum response factor (SRF; Protein-Tech custom); anti-smooth muscle α-actin (ACTA2; Sigma, clone 1A4); anti-integrin α5 (ITGA5; Millipore, AB1921); anti-fibronectin (FN1; Millipore, AB2033); anti-vinculin (VCL; abcam, ab18058); anti-BrdU (Invitrogen, 03-3900); anti-Histone H3, phosphorylated form (PhH3; Millipore 06-570); anti-vascular endothelial cadherin (CDH5, a.k.a. VE-Cad; abcam 33168); anti-epithelial cadherin (CDH1, a.k.a. E-Cad; BD Biosciences 610181). eYFP protein was detected using anti-GFP antibody (Invitrogen, A10262). Anti-ACTA2 antibody clone 1A4 detects α-actin found in several cell types, and was not used as a specific SMC label. DNA/nuclei were counterstained with DAPI. Terminal deoxynucleotide transferase dUTP nick end labeling (TUNEL) was performed on zinc formalin fixed paraffin-embedded 5 μm sections using the DeadEnd Colorimetric Apoptosis Detection System (Promega); DNase-treated wild-type VYS tissue was used as a positive control according to detection system protocol. Tissues harvested for detection of Tie2-Cre-associated β-galactosidase activity were fixed in 4% paraformaldehyde and LacZ stained under standard protocols. Digital image capture was performed using a Nikon Eclipse 80i microscope equipped with Nikon Digital Sight DS-2MBW monochrome and DS-F1 color cameras and NIS Elements-D imaging software. Tri-color image merge and post-processing was done in Adobe Photoshop CS4.

### Cytometric analysis

Thin sections (5 μm) of embryonic yolk sac from wild-type and *Tie2Cre^+/0^·Srf^f/f ^*embryos were stained for designated markers and imaged for cell counting. Positive nuclear staining was scored only in cells of the mesoderm layer of the visceral yolk sac. Between 300-500 nuclei over 5 visual fields (1 per section; 200× magnification) were counted for each embryo, with a total of 3 embryos per treatment group; embryos were taken from 2-3 different litters harvested on different days. Tissues were stained for SRF protein to verify loss of expression in BrdU treated tissues. Anti-BrdU antibody was used to detect incorporation into nuclear material of proliferating cells; PhH3 was used to label cells undergoing mitosis. Detection of ACTA2 protein was used to verify loss of *Srf *expression in PhH3 stained tissues due to incompatibility between anti-PhH3 and anti-SRF antibodies. DAPI was used as counter-stain and to identify all visible nuclei. Final cell counts of cells staining positive for SRF, BrdU or PhH3 were expressed as a percentage of total number of DAPI-positive nuclei counted.

### Real-time quantitative PCR (qPCR) and analysis

Embryos were harvested from timed pregnant females, and yolk sac tissues were dissected and processed for RNA (Qiagen RNeasy Mini Kit) and cDNA template (SABiosciences RT^2 ^First Strand Kit C-03) using published protocols. Samples were probed using SYBR Green with ROX reference (SABiosciences RT^2 ^Real-Time SYBR Green/Rox Master Mix PA-012) using 100 nm primer oligos under published protocols in a Stratagene Mx3005P Real-Time PCR System. Wild type and *Tie2Cre^+/0^·Srf^f/f ^*whole VYS tissues (n = 3 each) were assayed separately using 100 ng template per reaction. Differences between wild type and SRF null tissues were calculated using the 2^ΔΔCt method based on the Guide to Performing Relative Quantitation of Gene Expression Using Real-Time Quantitative PCR (Applied Biosystems) with all genes normalized to *Gapdh *expression. Data is presented as fold change with positive SEM only owing to the asymmetrical nature of confidence intervals in exponential fold change calculations. See Additional File 5 for real-time qPCR primer sequences.

## Authors' contributions

MLH carried out the immunofluorescence and qPCR studies and helped to draft the manuscript. RPM conceived of the study, participated in its design and coordination, and helped to draft the manuscript. Both authors read and approved the final manuscript.

## Supplementary Material

Additional file 1 **Tie2-Cre expression is restricted to VYS mesoderm**. Color photomicrographs of Lac-Z stained VYS tissues from *Tie2-Cre^+/0 ^*·*ROSA26R-βgal^+/+ ^*embryos in whole mount (A & B) and cross-sectional (C & D) views. We used *ROSA26R-β-galactosidase *reporter transgenic mice to examine the lineage distribution pattern of cells affected by Tie2-Cre recombinase activity. The *Tie2-Cre *construct begins expressing at E7.5 in endothelial cells and hematopoietic progenitor cells within blood islands of the visceral yolk sac (VYS) mesoderm where it remains until 9.5. Examination of whole mount tissues revealed robust signal in major vessels of VYS at E10.5 (A, arrow). By E12.5 this signal is widespread in VYS mesoderm (B, arrow). Cross-sectional analysis of VYS tissues at E10.5 (C) and E12.5 (D) demonstrate the strict confinement of Lac-Z signal to VYS mesoderm. Magnification C & D = 200×; scale bar D = 100 μm. Ed = endoderm, Md = mesoderm, * = blood vessel lumen. Click here for file

Additional file 2 **ACTA2 expression is disrupted prior to complete loss of SRF**. Double-label immunofluorescence analysis of SRF and ACTA2 expression in wild type (A & C) and *Tie2Cre^+/0^·Srf^f/f ^*(B & D) embryos at E10.5 and E11.5. Expression of ACTA2 is SRF-dependent and is apparent in wild-type visceral yolk sac tissues by E10.5 (A), and remains strong at E11.5 (C) and E12.5 (see Figure 2A). This robust level of protein decreases noticeably in SRF-null tissues by E10.5 when SRF levels are low but still detectable(B), suggesting that ACTA2 expression is acutely sensitive to regulation by SRF. Further decrease is observed at E11.5 (D). Magnification = 200×; scale = 50 μm. Ed = endoderm, Md = mesoderm. Click here for file

Additional file 3 **PhH3 analysis indicates SRF-null VYS mesoderm tissues show aberrant proliferation**. Immunodetection of PhH3 was used to assess proliferation in VYS mesoderm tissues. Double-label immunofluorescence images of VYS tissue from wild type (A, B) and SRF-null (C, D) embryos at E12.5. Tissues were stained for PhH3 (green), ACTA2 (red), and DNA (blue); monochrome images in B and D show PhH3 staining in isolation. Arrows in A and B highlight SRF-positive nuclei that colocalize with PhH3; arrowheads in C and D mark nuclei lacking SRF that stain positively for PhH3. Dashed line indicates division between Ed and Md layers. Magnification = 200×, scale bar = 50 μm; Ed = endoderm, Md = mesoderm. (C) Cytometric analysis of PhH3-positive nuclei in E10.5, E11.5 and E12.5 VYS mesoderm. Values are expressed as a percentage of total DAPI-stained nuclei counted. *p = 0.00005; **p = 0.025. Click here for file

Additional file 4 **Loss of SRF in VYS mesoderm does not alter the level of apoptosis**. Colorimetric TUNEL analysis in wild type (A) and *Tie2Cre^+/0^·Srf^f/f ^*(B) VYS tissues at E12.5 to detect DNA damage caused by apoptosis. DNase treatment of wild-type E12.5 VYS tissue was used as a positive control (C). We did not observe differences in the rate of apoptosis between wild-type and SRF-null tissues. Magnification = 200×, scale = 100 μm. Ed = endoderm, Md = mesoderm. Click here for file

Additional file 5 **Primers for qPCR**. Two independently published sets of qPCR primers for the *Ilk *gene were used in our analysis. Neither set generated any signal in any VYS tissue we assayed. Primer sets were tested for specificity against HeLa cell cDNA and successfully detected appropriate signal. Click here for file
