# Supplementary figures and images for: Serum response factor is required for cell contact maintenance but dispensable for proliferation in visceral yolk sac endothelium
Source: BMC Dev Biol. 2011 Mar 14;11:18. doi: 10.1186/1471-213X-11-18 (PMC3065428; doi:10.1186/1471-213X-11-18)

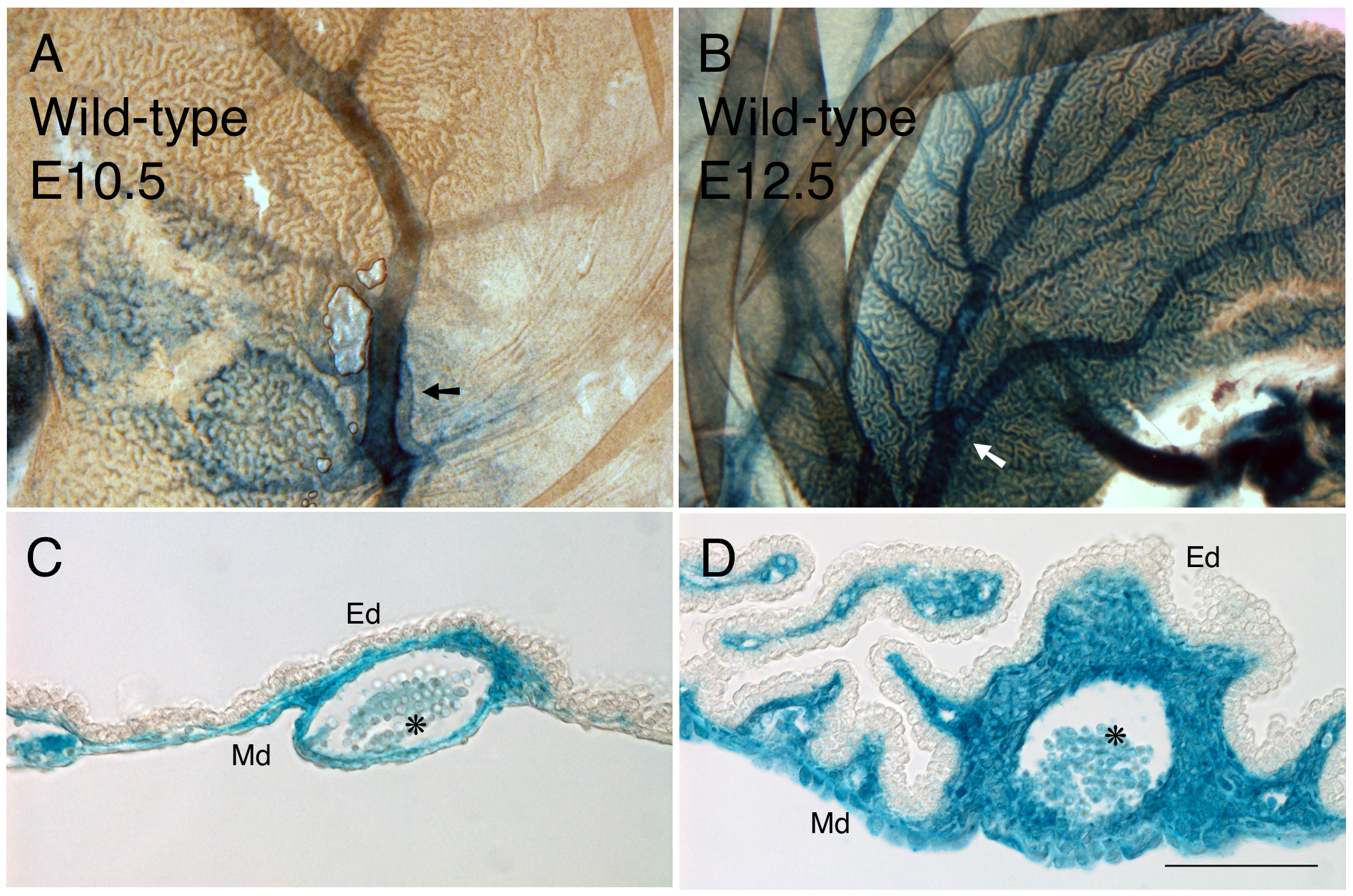

Supplement: Additional file 1 — Tie2-Cre expression is restricted to VYS mesoderm. Color photomicrographs of Lac-Z stained VYS tissues from Tie2-Cre+/0 ·ROSA26R-βgal+/+ embryos in whole mount (A & B) and cross-sectional (C & D) views. We used ROSA26R-β-galactosidase reporter transgenic mice to examine the lineage distribution pattern of cells affected by Tie2-Cre recombinase activity. The Tie2-Cre construct begins expressing at E7.5 in endothelial cells and hematopoietic progenitor cells within blood islands of the visceral yolk sac (VYS) mesoderm where it remains until 9.5. Examination of whole mount tissues revealed robust signal in major vessels of VYS at E10.5 (A, arrow). By E12.5 this signal is widespread in VYS mesoderm (B, arrow). Cross-sectional analysis of VYS tissues at E10.5 (C) and E12.5 (D) demonstrate the strict confinement of Lac-Z signal to VYS mesoderm. Magnification C & D = 200×; scale bar D = 100 μm. Ed = endoderm, Md = mesoderm, * = blood vessel lumen. [file 1471-213X-11-18-S1.PNG]

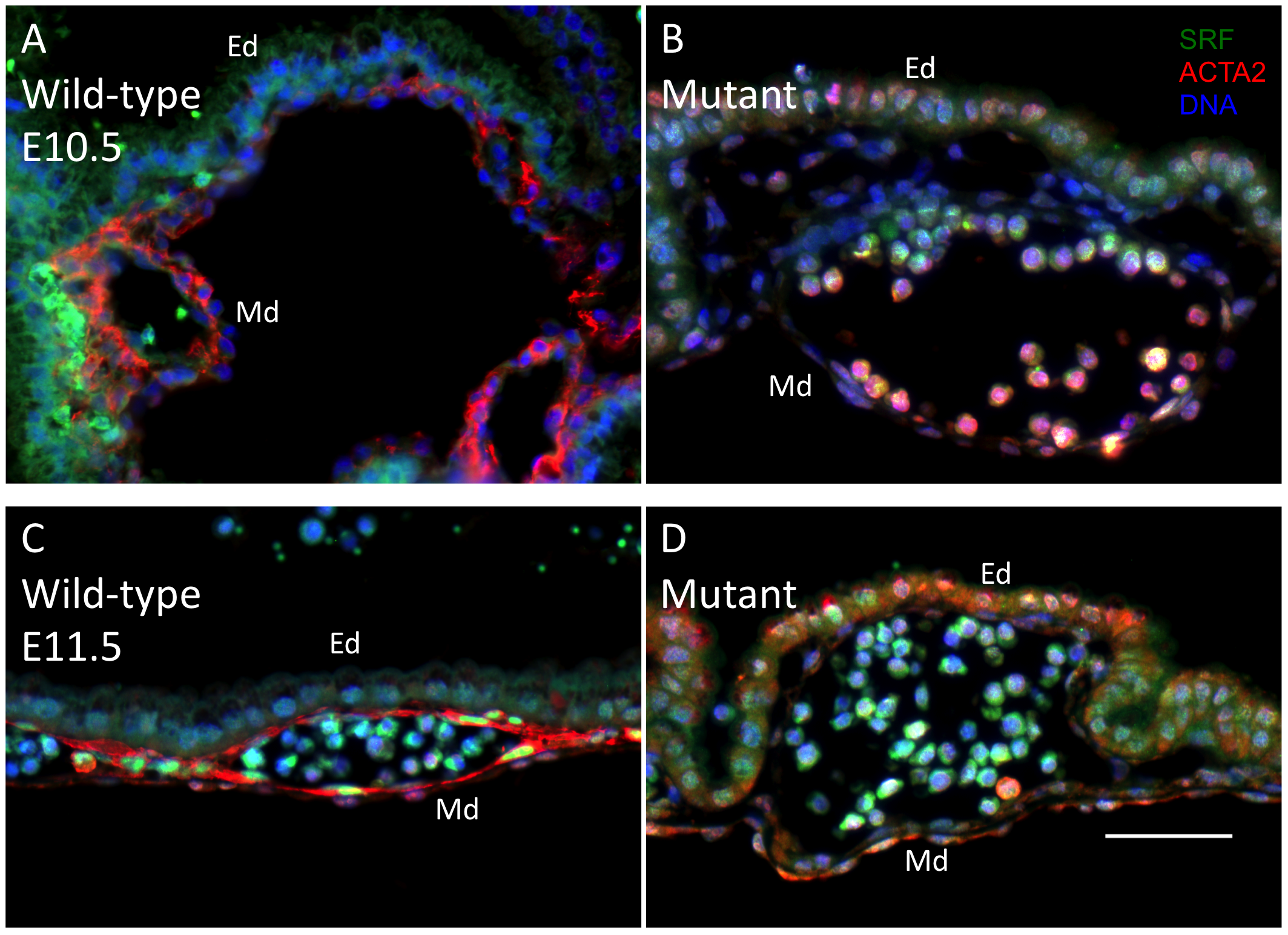

Supplement: Additional file 2 — ACTA2 expression is disrupted prior to complete loss of SRF. Double-label immunofluorescence analysis of SRF and ACTA2 expression in wild type (A & C) and Tie2Cre+/0·Srff/f (B & D) embryos at E10.5 and E11.5. Expression of ACTA2 is SRF-dependent and is apparent in wild-type visceral yolk sac tissues by E10.5 (A), and remains strong at E11.5 (C) and E12.5 (see Figure 2A). This robust level of protein decreases noticeably in SRF-null tissues by E10.5 when SRF levels are low but still detectable(B), suggesting that ACTA2 expression is acutely sensitive to regulation by SRF. Further decrease is observed at E11.5 (D). Magnification = 200×; scale = 50 μm. Ed = endoderm, Md = mesoderm. [file 1471-213X-11-18-S2.PNG]

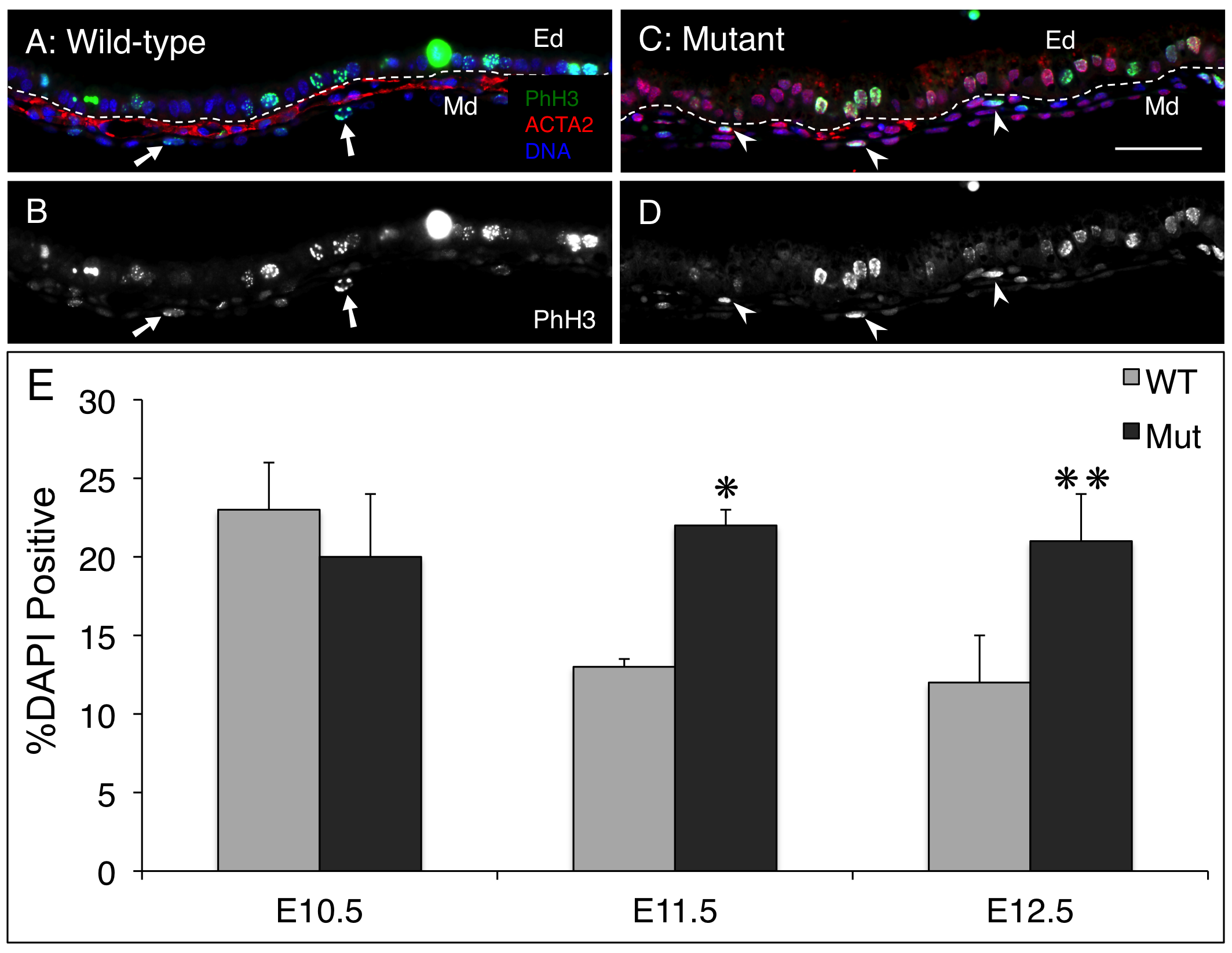

Supplement: Additional file 3 — PhH3 analysis indicates SRF-null VYS mesoderm tissues show aberrant proliferation. Immunodetection of PhH3 was used to assess proliferation in VYS mesoderm tissues. Double-label immunofluorescence images of VYS tissue from wild type (A, B) and SRF-null (C, D) embryos at E12.5. Tissues were stained for PhH3 (green), ACTA2 (red), and DNA (blue); monochrome images in B and D show PhH3 staining in isolation. Arrows in A and B highlight SRF-positive nuclei that colocalize with PhH3; arrowheads in C and D mark nuclei lacking SRF that stain positively for PhH3. Dashed line indicates division between Ed and Md layers. Magnification = 200×, scale bar = 50 μm; Ed = endoderm, Md = mesoderm. (C) Cytometric analysis of PhH3-positive nuclei in E10.5, E11.5 and E12.5 VYS mesoderm. Values are expressed as a percentage of total DAPI-stained nuclei counted. *p = 0.00005; **p = 0.025. [file 1471-213X-11-18-S3.PNG]

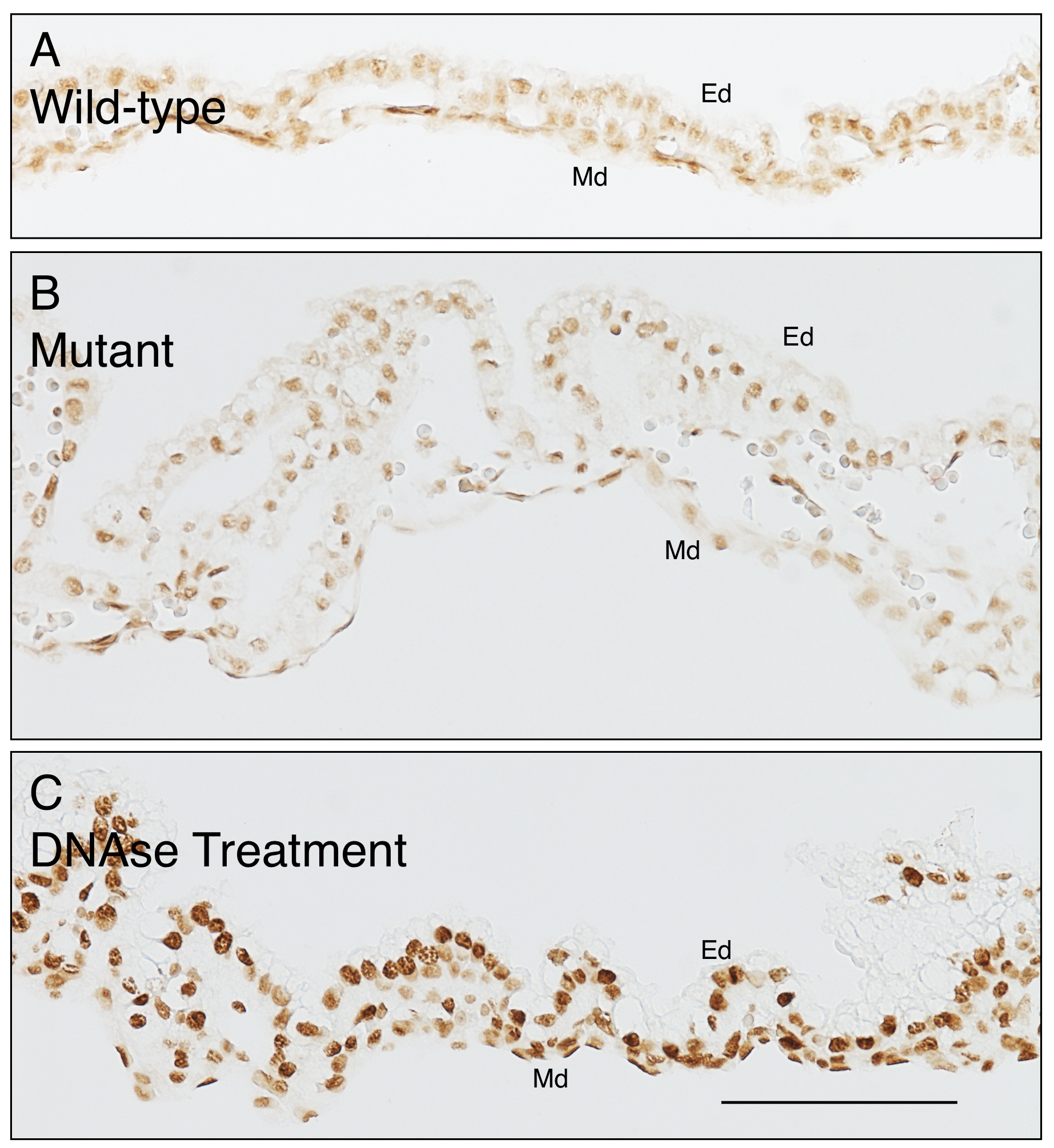

Supplement: Additional file 4 — Loss of SRF in VYS mesoderm does not alter the level of apoptosis. Colorimetric TUNEL analysis in wild type (A) and Tie2Cre+/0·Srff/f (B) VYS tissues at E12.5 to detect DNA damage caused by apoptosis. DNase treatment of wild-type E12.5 VYS tissue was used as a positive control (C). We did not observe differences in the rate of apoptosis between wild-type and SRF-null tissues. Magnification = 200×, scale = 100 μm. Ed = endoderm, Md = mesoderm. [file 1471-213X-11-18-S4.PNG]
