# Supplementary material for: Serum response factor is required for cell contact maintenance but dispensable for proliferation in visceral yolk sac endothelium
Source: BMC Dev Biol. 2011 Mar 14;11:18. doi: 10.1186/1471-213X-11-18 (PMC3065428; doi:10.1186/1471-213X-11-18)
Supplement: Additional file 5 — Primers for qPCR. Two independently published sets of qPCR primers for the Ilk gene were used in our analysis. Neither set generated any signal in any VYS tissue we assayed. Primer sets were tested for specificity against HeLa cell cDNA and successfully detected appropriate signal. [file 1471-213X-11-18-S5.PDF]

Target gene	Symbol	Primer	Sequence	Source
Gapdh	*Gapdh*		NA	SABiosciences catalog #PPM02946E
Serum response factor	*Srf*		NA	SABiosciences catalog #PPM03580E
β-Actin	*Actb*	Fwd	gat ctg gca cca cac ctt ct	Franco et al., 2008
		Rvs	ggg gtg ttg aag gtc tca aa	
Smooth muscle α-actin	*Acta2*	Fwd	tgt gct gga ctc tgg aga tg	Franco et al., 2008
		Rvs	gaa gga ata gcc acg ctc ag	
PECAM1	*Pecam1*	Fwd	gct gct cca ctt ctg aac tcc	Holtz and Misra
		Rvs	gca tca ttt cca gaa aca tca tca taa cc	
VE-Cadherin	*Cdh5*	Fwd	cca tct tcc tct gca tcc tc	Franco et al., 2008
		Rvs	caa ctg ctc gtg aat ctc ca	
β-Catenin	*Ctnnb1*	Fwd	atg gag ccg gac aga aaa gc	Cadoret et al., 2001
		Rvs	ctt gcc act cag gga agg a	
Claudin 5	*Cldn5*	Fwd	gca agg tgt atg aat ctg tgc t	PrimerBank 31980735a1
		Rvs	gtc aag gta aca aag agt gcc a	
Tight junction protein 1 (a.k.a. ZO1)	*Tjp1*	Fwd	agc aag cct tct gca cat ct	Franco et al., 2008
		Rvs	cag cat cag ttt cgg gtt tt	
Integrin α5	*Itga5*	Fwd	caa ggt gac agg act cag ga	Franco et al., 2008
		Rvs	ggt ctc tgg atc caa ctc ca	
Integrin β1	*Itgb1*	Fwd	tca cat gca ggt ttg gaa aa	Franco et al., 2008
		Rvs	tgt gac ctc agc tga caa gg	
Vinculin	*Vcl*	Fwd	tgg acg gca aag cca ttc c	Chen et al., 2009
		Rvs	gct ggt ggc ata tct ctc ttc ag	
Fibronectin 1	*Fn1*	Fwd	atg tgg acc cct cct gat agt	PrimerBank 1181242a1
		Rvs	gcc cag tga ttt cag caa agg	
Integrin linked kinase set A*	*ILK*	Fwd	cat caa tgc agt gaa tga gc	Chen et al., 2008
		Rvs	gac att cct cat tga agt cc	
Integrin linked kinase set B*	*ILK*	Fwd	atg aga atc att ctg gag agc ttt g	El-Aouni et al., 2006
		Rvs	tgt act cca gtc tcg aac ctt cag	

* Two independently published sets of qPCR primers for the *Ilk* gene were used in our analysis. Neither set generated any signal in any VYS tissue we assayed. Primer sets were tested for specificity against HeLa cell cDNA and successfully detected appropriate signal.

Cadoret A, Ovejero C, Saadi-Kheddouci S, Souil E, Fabre M, Romagnolo B, Kahn A, Perret C. **Hepatomegaly in transgenic mice expressing an oncogenic form of β-catenin.** 2001 *Canc. Res.* 61:3245-3249.

Chen J, Gui D, Chen Y, Mou L, Liu Y, Huang J. **Astragaloside IV improves high glucose-induced podocyte adhesion dysfunction via α3β1 integrin upregulation and integrin-linked kinase inhibition.** 2008 *Biochem. Pharmacol.* 76: 796-804.

Chen S, Wang R, Li QF, Tand DD. **Ab1 knockout differentially affects p130 Crk-associated substrate, vinculin, and paxillin in blood vessels of mice.** 2009 *Am. J. Physiol. Heart Circ. Physiol.* 297:H533-H539.

El-Aouni C, Herbach N, Blattner SM, Henger A, Rastaldi MP, Jarad G, Miner JH, Moeller MJ, St-Arnaud R, Dedhar S, Holzman LB, Wanke R, Kretzler M. **Podocyte-specific deletion of integrin-linked kinase results in severe glomerular basement membrane alterations and progressive glomerulosclerosis.** 2006 *J. Am. Soc. Nephrol.* 17: 1334-1344.

Franco CA, Mericskay M, Parlakian A, Gary-Bobo G, Gao-Li J, Paulin D, Gustafsson E, Li Z. **Serum response factor is required for sprouting angiogenesis and vascular integrity.** 2008 *Dev. Cell* 15:448-461.
